# Plant polycistronic precursors containing non-homologous microRNAs target transcripts encoding functionally related proteins

**DOI:** 10.1186/gb-2009-10-12-r136

**Published:** 2009-12-01

**Authors:** Francisco Merchan, Adnane Boualem, Martin Crespi, Florian Frugier

**Affiliations:** 1 Institut des Sciences du Végétal (ISV), Centre National de la Recherche Scientifique (CNRS), Avenue de la terrasse, 91198 Gif sur Yvette cedex, France; 2 Current address: Dto Microbiología y Parasitología, Facultad de Farmacia, Universidad de Sevilla, C/Profesor García González 2, 41012 Sevilla, Spain; 3 Unité de recherche en Génomique Végétale (URGV), Institut National de la Recherche Agronomique (INRA), Rue Gaston Crémieux, 91057 Evry cedex, France

## Abstract

**Background:**

MicroRNAs (miRNAs) are endogenous single-stranded small RNAs that regulate the expression of specific mRNAs involved in diverse biological processes. In plants, miRNAs are generally encoded as a single species in independent transcriptional units, referred to as *MIRNA *genes, in contrast to animal miRNAs, which are frequently clustered.

**Results:**

We performed a comparative genomic analysis in three model plants (rice, poplar and *Arabidopsis*) and characterized miRNA clusters containing two to eight miRNA species. These clusters usually encode miRNAs of the same family and certain share a common evolutionary origin across monocot and dicot lineages. In addition, we identified miRNA clusters harboring miRNAs with unrelated sequences that are usually not evolutionarily conserved. Strikingly, non-homologous miRNAs from the same cluster were predicted to target transcripts encoding related proteins. At least four *Arabidopsis *non-homologous clusters were expressed as single transcriptional units. Overexpression of one of these polycistronic precursors, producing Ath-miR859 and Ath-miR774, led to the DCL1-dependent accumulation of both miRNAs and down-regulation of their different mRNA targets encoding F-box proteins.

**Conclusions:**

In addition to polycistronic precursors carrying related miRNAs, plants also contain precursors allowing coordinated expression of non-homologous miRNAs to co-regulate functionally related target transcripts. This mechanism paves the way for using polycistronic *MIRNA *precursors as a new molecular tool for plant biologists to simultaneously control the expression of different genes.

## Abstract 2

Functional homologous and non-homologous clusters of MIR genes that co-regulate target mRNA transcripts have been identified in plants

## Background

MicroRNAs (miRNAs) are endogenous approximately 21-nucleotide single-stranded small RNAs derived from *MIRNA *precursors that are able to fold-back into a stable secondary structure (stem loop or hairpin). miRNAs act in many developmental processes as well as environmental and pathogenic responses [1-4] through the post-transcriptional regulation of target mRNAs. These targets carry a sequence-specific miRNA recognition site, leading to transcript cleavage and/or inhibition of mRNA translation [1,5,6]. Primary miRNA transcripts (pri-*MIRNA*) are transcribed by RNA polymerase II, and several ribonucleoprotein (RNP) complexes are involved in their maturation, a process that differs between animals and plants [1,6-11]. In animals, formation of an approximately 21-bp miRNA-miRNA* duplex successively involves two RNase III enzymatic complexes: the Drosha enzyme, which cleaves long pri-*MIRNA *in the nucleus to generate short (approximately 70- to 80-nucleotide) hairpins (so called pre-*MIRNA*) and the Dicer enzyme, which produces the miRNA after cytoplasmic export of pre-*MIRNA*s through Exportin 5 [11]. In plants, however, both cleavages are likely nuclear localized and involve a single Dicer-like enzyme 1 (DCL1) complex [6,9,10]. The miRNA-miRNA* duplex is exported to the cytoplasm by HASTY, the plant ortholog of Exportin 5 [12,13]. Subsequently, these duplexes are converted into single-stranded miRNAs upon incorporation into an ARGONAUTE (AGO) ribonucleoprotein complex, referred to as the RNA-induced silencing complex (RISC). The miRNAs guide sequence-specific cleavage and/or translational repression of target transcripts into the RISC complex [6,9-11].

Recent deep sequencing of plant small RNA libraries has led to the identification of more than 1,300 miRNAs in various plants (miRBase, release 13.0, March 2009) [14]. Based on comparison of all available plant genomes (even partial ones; 16 genera referenced in miRBase), evolutionarily conserved and non-conserved miRNAs have been proposed. The non-conserved miRNAs have probably emerged in recent evolutionary time scales, and show a wide diversity compared to the restricted number of conserved miRNAs [15]. Indeed, only 5 miRNA families are found in more than 40 plant species whereas 25 exist in more than one plant genus [16]. The three higher plant models showing the most comprehensive description of their miRNome are rice (*Oryza sativa*; 377 *MIRNA*s), poplar (*Populus trichocarpa*; 234 *MIRNA*s) and *Arabidopsis *(*Arabidopsis thaliana*; 187 *MIRNA*s), with 22 families 'conserved' between them (indicated in bold in Additional data file 1 based on miRBase 13.0). The numerous non-conserved miRNAs are thus likely to play species-specific roles [15].

Plant and animal *MIRNA *genes differ in their genomic location and organization. Most plant miRNAs are encoded in intergenic loci, whereas animal miRNAs are also frequently encoded within introns of protein coding genes [17-19]. Plant miRNAs are mainly generated from independent transcriptional units, whereas in *Drosophila*, nematodes, zebrafish and mammals, around 40 to 50% of the predicted *MIRNA *genes are located within clusters that are often evolutionarily conserved [18-27]. A maximal distance of 3 kb between two consecutive miRNAs has been used as a stringent criterion to estimate cluster numbers [18]. Clusters in animal genomes usually encode two to three miRNAs but some encode up to eight. Even larger miRNA clusters were predicted in human and zebrafish, containing more than 40 *MIRNA *loci [18,25,26]. In these clusters, miRNAs are encoded either in independent hairpins or sometimes in both arms of the same hairpin [28]. In plants, even though no systematic analysis of miRNA clusters has been performed in the different available genomes, a few miRNA clusters have been reported [16,29-33].

Clustered miRNAs can be either simultaneously transcribed into a single polycistronic transcript or independently transcribed [1,28,34]. Short distances between consecutive *MIRNA *loci and coordinated expression of clustered miRNAs are hallmarks of polycistronic transcription [18,22,34]. Most of the few reported plant miRNA clusters contain several copies of the same conserved miRNA (miR156, miR166, miR169, miR395 or miR399), in contrast to animals where miRNAs with unrelated sequences are often included in the same clusters [18,19,25,35]. Interestingly, certain animal miRNA clusters showing co-regulated expression can simultaneously target transcripts encoding different functionally related proteins. It has been proposed that this may coordinate the fine tuning of the regulation of specific molecular processes [1,18,19,25]. Recently, functional analysis of two human miRNA clusters revealed that the different encoded miRNAs co-regulate related cyclin dependent kinase inhibitors and facilitate cell cycle progression [27]. In plants, beyond the identification of a few expressed sequence tags (ESTs) spanning miRNA clusters [16,29-33], few experimental data indicate that clustered miRNAs are transcribed simultaneously. In the model legume *Medicago truncatula*, a miR166 tandem was shown to be encoded in a single transcriptional unit [32]. However, as both miRNAs are nearly identical, it is difficult to definitively conclude that this pri-*MIRNA *generates more than one miRNA.

In this study, we demonstrate that approximately 20% of plant miRNAs are clustered, and generally contain conserved miRNAs of the same family. Synteny analysis suggested a common evolutionary origin for certain clusters. Strikingly, a few clusters encode tandem non-conserved miRNAs with unrelated sequences, whose predicted targets correspond to transcripts encoding related proteins. In *Arabidopsis*, four of these clusters were transcribed as polycistronic precursors and we show that at least one cluster is processed to form both mature miRNA species in a DCL1-dependent manner. Accumulation of the mature miRNAs affected the stability of their respective predicted target transcripts. Consequently, plant polycistronic *MIRNA *precursors can encode functional non-homologous miRNAs. This genomic organization may serve to co-regulate different mRNA targets post-transcriptionally.

## Results

### *In silico *identification of miRNA clusters in *Arabidopsis*, rice and poplar genomes

A systematic search for consecutive *MIRNA *loci was carried out in three model plant genomes that have an exhaustive description of their miRNA species (miRBase 13.0 [14]). Initially, a 3-kb distance between consecutive *MIRNA *was used as a stringent criterion to define miRNA clusters, similar to previous studies in animals [18,26]. As a result, 16, 10 and 9 clusters were identified in rice, *Arabidopsis*, and poplar, respectively, which represented 13%, 11% and 8% of the total *MIRNA *loci (Table 1; Additional data file 2). Co-expression studies and ESTs available in animal genomes have indicated that some miRNA clusters can be very large; therefore, the 3-kb criterion, which is useful to avoid overestimation of miRNA clusters, is probably too stringent [18,25,35]. Using a less stringent 10-kb cluster size criterion, the number of plant miRNA clusters increased to 18 to 24 in these genomes, thus representing up to 22% of the total *MIRNA *loci (Table 1).

**Table 1:** **Summary of clustered miRNAs in *Arabidopsis thaliana*, rice (*Oryza sativa*) and poplar (*Populus trichocarpa*) genomes**2

	*Populus trichocarpa*	*Oryza sativa*	*Arabidopsis thaliana*
Number of clusters with consecutive miRNAs at a distant of (same strand miRNAs):			
<1 kb	9 (9)	16 (14)	10 (8)
<3 kb	14 (14)	16 (14)	13 (11)
<10 kb	20 (20)	24 (22)	18 (16)
			
% of clustered miRNAs (number of clustered miRNAs/total miRNAs)	20% (47/234)	19% (68/353)	22% (42/187)
			
Maximal number of miRNAs encoded in a cluster (cluster size)	4 (4,593 bp)	8 (497 bp)	6 (5,522 bp)
			
% of clusters with (number/total of <10 kb miRNA clusters):			
A single miRNA family	90% (18/20)	75% (18/24)	61% (11/18)
Conserved miRNAs	90% (18/20)	54% (13/24)	44% (8/18)

Analyses were based on miRNA described in miRBase version 13.0 (March 2009).

**Figure 1:**
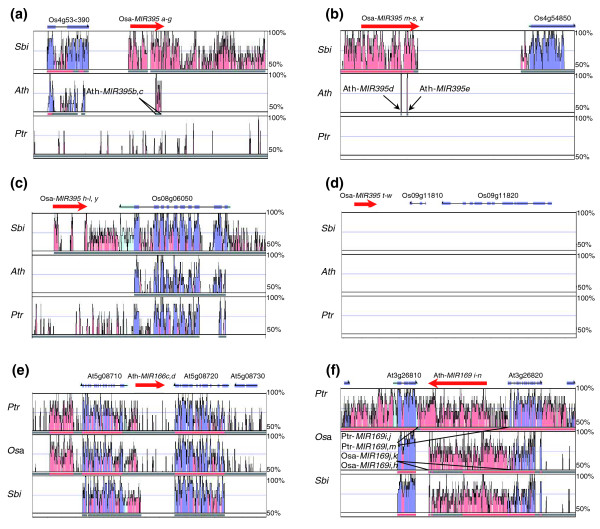
Microsynteny between homologous miRNA clusters conserved between *A. thaliana*, rice (*O. sativa*) and poplar (*P. trichocarpa*). VISTA plots [37,70] shows the conservation of different clustered miRNAs in the three selected genomes (Table 1; Additional data file 2): **(a-d) **the four rice miR395 clusters; **(e) **the Ath-*MIR166c*, *d *cluster; **(f) **the Ath-*MIR169i-n *cluster. To analyze evolutionary conservation between monocots, the sorghum genome is indicated. In each graph, gene models (blue for protein coding gene exons, and red for miRNA clusters) are indicated above, and percentage (50 to 100%) on the right side indicates the level of identity between target and reference genomes, visualized through pink and blue peaks for intronic/non-protein coding, and exonic regions, respectively. The name of syntenic clusters in non-reference genomes is indicated, and syntenic clusters are delimited by black bars. Ath, *Arabidopsis thaliana*; Ptc, *Populus trichocarpa*; Osa, *Oryza sativ*a, Sbi, *Sorghum bicolor*.

Independently of the size threshold used, most of the clusters (61%, 75% and 90% in *Arabidopsis*, rice and poplar, respectively) contained several copies of the same miRNA family, generally two to three and a maximum of eight (the latter is the rice Osa-*MIR395m-s*, *x *cluster spanning 497 bp; Table 1; Additional data file 2), and were therefore called homologous clusters. These clusters frequently contained conserved miRNAs (Additional data file 1), and represent 90%, 54% and 44% of the clustered miRNAs in poplar, rice, and *Arabidopsis*, respectively (Table 1). Homologous clusters were found for miR166, miR169 and miR395 families (based on the <10-kb threshold). This suggested a putative common origin of these clusters, involving successive gene duplications and losses as described for animal miRNA clusters [18,28]. Analysis of these miRNA clusters using VISTA Plot [36,37] (Figure 1) revealed that some were syntenic between monocot and dicot plants (Figure 1a, b, f; two rice miR395 clusters and the Ath-*MIR169i-n *locus) or only within monocot plants (Figure 1c; a third rice miR395 cluster). Surprisingly, no miR395 syntenic locus could be retrieved in the poplar genome (Figure 1a-d). Other miRNA clusters were specific to one plant genome analyzed (Figure 1d, e; the fourth rice miR395 cluster and the Ath-*MIR166c*, *d *locus). These results suggest that certain ancestral miRNA clusters appeared before the divergence of monocot and dicot lineages and showed differential expansions in the various plant genomes analyzed. Furthermore, clustering of specific miRNAs (for example, miR395, miR169, miR166) is evolutionarily conserved.

### Non-homologous miRNAs are expressed as polycistronic pri-*MIRNAs*

**Figure 2:**
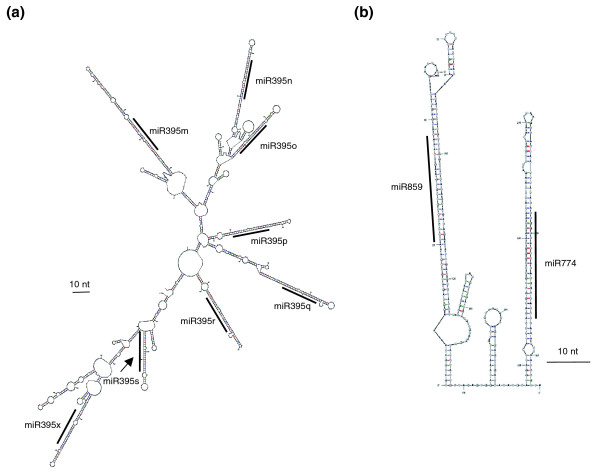
Representative RNA secondary structure of putative polycistronic clustered miRNAs. Representative examples of **(a) **a homologous miRNA cluster, Osa-*MIR395m-s*, *x*, and **(b) **a non-homologous cluster, Ath-*MIR859-774*. Mfold software [66,67] was used to generate the most probable RNA secondary structures. Mature miRNA sequences are indicated with a line. In the case of the rice cluster, a new miR395 locus (Osa-*MIR395x*) not yet present in miRBase (version 13.0) was annotated. Ath, *Arabidopsis thaliana*; Osa, *Oryza sativ*a. Black bar = 10 nucleotides (nt).

When *MIRNA *loci at a distance of <1 kb on the same DNA strand were considered, 8, 9 and 14 miRNA clusters were identified in *Arabidopsis*, poplar, and rice, respectively (Table 1). In contrast, only four clusters encoding two miRNAs on opposite strands were found (Additional data file 2). These results indicate that short range clustering (based on the 1-kb threshold) is strongly biased (χ^2 ^test, *P *= 9.4 E-13) towards 'same DNA-strand' clustering, suggesting an eventual co-transcription. As small clusters may correspond to polycistronic *MIRNA *precursors, we searched pri-*MIRNA*s containing several tightly linked miRNAs (Additional data file 2). For example, homologous rice miR395 clusters show the highest number of miRNAs, each encoded in independent stem-loops that are probably generated by successive duplications of an ancestral hairpin (Figure 2a; Figure S1 in Additional data file 3). Folding analyses also revealed additional hairpins in the rice Osa-*MIR395m-s *and Osa-*MIR395h-l *clusters containing new miR395 loci not yet listed in miRBase (Osa-*MIR395x *[MiRBase:MI0013350] and Osa-*MIR395y *[MiRBase:MI0013351]; Additional data file 2).

In addition to clusters encoding homologous miRNAs, several clusters (two in rice, three in poplar and five in *Arabidopsis*) consisting of miRNAs with unrelated sequences were identified (Table 2). These non-homologous clusters mainly corresponded to non-conserved miRNAs, and their size ranged between 271 and 1,192 bp, with predicted hairpins separated by 81 to 670 bp (median of 114 bp; Additional data file 2), strongly suggesting that these clusters were polycistronic. Most of them were encoded in regions located between protein-coding genes, with the exception of two rice clusters that derive from intronic regions of different putative ribosomal protein encoding genes (ESTs listed in Table 2). In most cases, non-homologous clusters encoded two miRNAs, with the exception of the Osa-*MIR1876-1862d-1884b *cluster. Similarly to the homologous miRNA clusters, this novel *MIRNA *precursor class contained several hairpins, each carrying a single annotated miRNA in the stem (Figure 2b; Figures S2 and S3 in Additional data file 3). A majority of 21- to 22-bp species was found for each predicted miRNA in *Arabidopsis *non-homologous clusters (Genome View browser, *Arabidopsis *Small RNA Project (ASRP) database) [38-40], and we systematically annotated miRNA* to identify *bona fide *miRNAs [41] (Figure S4 in Additional data file 3). However, for Ath-*MIR859*, Ath-*MIR397b *and Ath-*MIR857 *loci, no miRNA* could be identified in small RNA databases.

**Table 2:** Summary of targets predicted for non-homologous putative polycistronic clustered miRNAs in *Arabidopsis*, rice and poplar

		Targets	
		
Non-homologous *MIRNA *clusters	ESTs overlapping the cluster	Predicted	Cleavage validated for at least one target [reference]*
*Arabidopsis*			
Ath-*MIR397b-857*	No	Laccases (3/1)	Yes [15,49,50]
Ath-*MIR859-774*	No	F-box proteins (35/5)	Yes [15,48]
Ath-*MIR842-846*	No	JR/MBP (1/10)	Yes [15]
Ath-*MIR851-771*	No	EIF2 for miR771 (1) no target for miR851	No [15]
Ath-*MIR850-863*	EG495879 EG495880 EG514601 DR380439, AV523115^†^	No target	-
Poplar			
Ptc-*MIR482-1448*	DB893204 DB893031 BP928209 CX175581	Disease resistance proteins (12/2)	Yes [44]
Ptc-*MIR1446b-477a*	No	Gibberellin response modulator-like protein (DELLA) (1)	Yes [43,44]
Ptc-*MIR1446a-477b*	No	Gibberellin response modulator-like protein (DELLA) (1)	Yes [43,44]
Rice			
Osa-*MIR1876-1862d-1884b*	Os10g0170200^‡^	No target	-
Osa-*MIR1423-1868*	Os04g32710^‡^	No target	-

For predicted targets, the number of targets found for each miRNA of the non-homologous cluster is indicated in parentheses. *Validated using 5' RACE (rapid amplification of cDNA ends) PCR. ^†^ESTs cover the region including only miR863. ^‡^The consecutive miRNAs are predicted in the intron of a protein coding gene (putative ribosomal protein). Ath, *A. thaliana*; Osa, *O. sativa*; Ptc, *P. trichocarpa*.

**Figure 3:**
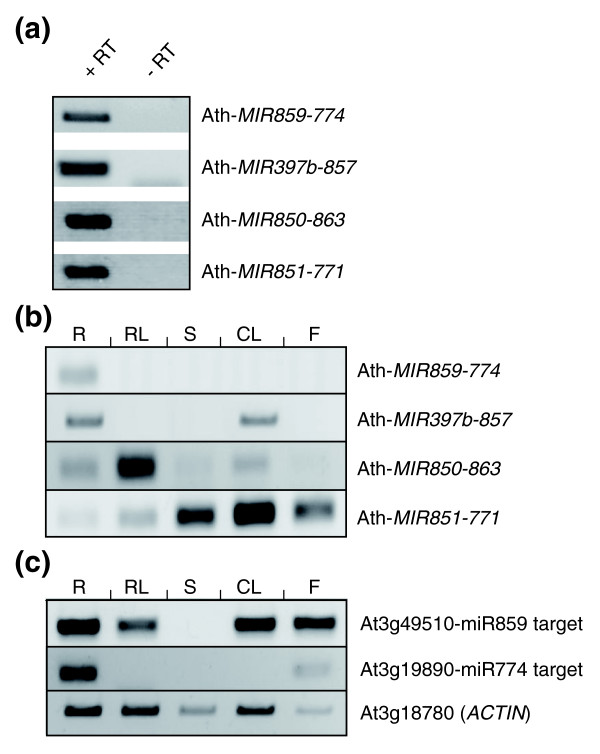
Expression of *Arabidopsis *polycistronic non-homologous miRNA clusters and selected targets in different organs. **(a) **Detection by RT-PCR analysis of the expression of *Arabidopsis *polycistronic non-homologous clusters as single transcriptional units: Ath-*MIR859-774*, Ath-*MIR397b-857*, Ath-*MIR850-863*, and Ath-*MIR851-771*. Total RNAs from wild-type (Col-0) seedlings were used for DNAse I treatment and cDNA synthesis to perform RT-PCR reactions. A control without reverse transcriptase (-RT) was systematically included to check for the absence of genomic DNA. Specificity of PCR amplicons was verified by sequencing. **(b) **Expression analysis by RT-PCR of the different *Arabidopsis *polycistronic non-homologous clusters (Ath-*MIR859-774*, Ath-*MIR397b-857*, Ath-*MIR850-863*, and Ath-*MIR851-771*) in different organs (R, roots; RL, rosette leaves; S, stems; CL, cauline leaves; F, flowers). **(c) **Expression analysis by RT-PCR of selected Ath-*MIR859-774 *targets in the same organs (roots, rosette or cauline leaves, stems, flowers) as in (b). The *Arabidopsis *Information Resource database entry TAIR:At3g49510 was used as an Ath-miR859 validated target (by 5' RACE (rapid amplification of cDNA ends) PCR) [15], and TAIR:At3g19890 for Ath-miR774 [48]. At3g18780 encoding an *ACTIN *isoform was used as RNA loading control.

Currently, the *Arabidopsis *genomic regions corresponding to candidate polycistronic non-homologous *MIRNA *genes are represented as independent transcriptional units in the ASRP database (Figure S4 in Additional data file 3). Indeed, no EST comprising tandem miRNAs was available in *Arabidopsis*. ESTs spanning the two *MIRNA *hairpins were reported only for the poplar Ptc-*MIR482-1448 *locus (Table 2), indicating that these two miRNAs are indeed co-transcribed. To determine whether *Arabidopsis *non-homologous miRNA clusters are encoded into single polycistronic units, WWwe performed semi-quantitative RT-PCR experiments (Figure 3). Primers designed to amplify precursors spanning the two predicted hairpins revealed expression for Ath-*MIR859-774*, Ath-*MIR850-863*, Ath-*MIR851-771 *and Ath-*MIR397b-857 *in seedlings (Figure 3a; Figure S4 in Additional data file 3, with RT-PCR amplified regions indicated as ESTs with green arrows). Cloning and subsequent sequencing of RT-PCR products confirmed the specific amplification of these pri-*MIRNA*s ([NCBI:GU125419] for Ath-*MIR859-774*; [NCBI:GU125420] for Ath-*MIR850-863*; [NCBI:GU125421] for Ath-*MIR397B-857*; [NCBI:GU125422] for Ath-*MIR851-771*). However, no amplification was obtained for the Ath-*MIR842-846 *cluster (even using various primer combinations). Expression of these four polycistronic miRNAs in different *Arabidopsis *organs revealed specific patterns: Ath-*MIR859-774 *pri-*MIRNA *was only detectable in roots, Ath-*MIR397b-857 *in roots and cauline leaves, Ath-*MIR851-771 *in aerial parts, including flowers, and Ath-*MIR850-863 *mainly in rosette leaves (Figure 3b).

Overall, these results suggest that clusters comprising functional miRNAs with unrelated sequences exist in plants as single transcriptional units, defining a novel class of plant pri-*MIRNA *genes.

### Polycistronic non-homologous miRNAs regulate related targets

In contrast to animals, *in silico *predictions revealed only a few targets for each plant miRNA based on strong sequence complementarity [42]. Strikingly, all predicted targets corresponding to different miRNAs from the same cluster encode proteins of the same family (Table 2; complete target list in Additional data file 4; based on the ASRP database for *Arabidopsis*, and on [43-45] for poplar). Indeed, the Ath-*MIR397b-857 *locus encodes two miRNAs that regulate laccases (three and one gene, respectively), the Ath-*MIR842-846 *locus encodes two miRNAs that regulate JR/MBP proteins (Jacalin repeat/Myrosinase binding protein; one and ten genes, respectively), and the Ath-*MIR859-774 *locus encodes two miRNAs that regulate F-box proteins (35 and 5 genes, respectively). More importantly, three of these F-box proteins are likely to be targeted by both Ath-miR859 and Ath-miR774 (Additional data file 4). Similarly, the Ptc-*MIR482-1448 *locus encodes miRNAs that regulate disease resistance proteins (12 and 2 genes, respectively, based on various gene models [46]), and one of them is probably co-regulated by the two miRNAs (Additional data file 4). Finally, the two Ptc-*MIR1446-477 *loci encode miRNAs that both target a single 'gibberellin response modulator-like protein' homologous to the *Arabidopsis *RGL1/RGL2 (Repressor of gibberellic acid requiring (GA1)-LIKE; DELLA transcription factors [45]). For the other non-homologous clustered miRNAs, targets were either predicted for only one miRNA of the tandem (for example, a single EIF2 encoding transcript - The *Arabidopsis *Information Resource database entry TAIR:At1g76810 - for Ath-miR771), or no target could be identified (Table 2). Target validation based on 5' RACE (5' rapid amplification of cDNA ends) PCR experiments was determined in *Arabidopsis *(AtPARE database) [47] for Ath-miR859 [TAIR:At3g49510] [15], Ath-miR774 [TAIR:At3g19890] [48], Ath-miR397 ([TAIR:At2g29130], [TAIR:At5g60020] and [TAIR:At2g38080]) [49,50], Ath-miR857 [TAIR:At3g09220], Ath-miR842 [TAIR:At5g38550] and Ath-miR846 [TAIR:At5g49850] [15] (Table 2). In poplar, Ptc-miR477/Ptc-miR1446-mediated cleavage of the DPTF:fgenesh4_pg.C_LG_XII000915 target (from the Database of Poplar Transcription Factors) was validated for each miRNA, as well as the JGI-Ptr-v1.1:eugene3.00102261 target for Ptc-miR482 and JGI-Ptr-v1.1:eugene3.01310091 for Ptc-miR1448 (both from the Join Genome Institute poplar database) [43,44,46].

These analyses suggest that non-homologous miRNA polycistronic clusters are likely to target transcripts encoding proteins of the same family, suggesting that co-transcription of miRNAs may co-regulate their respective targets.

### The polycistronic Ath-*MIR859-774 *pri-MIRNA is processed by a DCL1-dependent pathway

**Figure 4:**
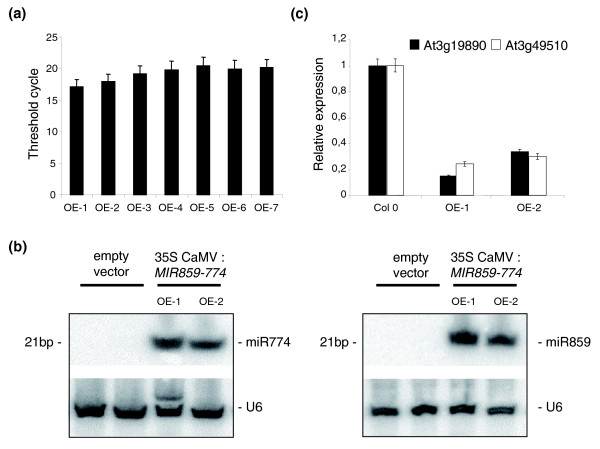
Ath-*MIR859-774 *encodes a functional polycistronic pri-*MIRNA*. **(a) **Overexpression of Ath-*MIR859-774 *pri-*MIRNA *(under the control of a 35S-CaMV promoter) in wild-type Col-0 transgenic lines (seven representative OverExpressing (OE) lines) is shown. Real-time RT-PCR analysis was performed based on equal amounts of total RNAs. A green fluorescent protein encoding transcript present 3' to the overexpressed pri-*MIRNA *was used as a tag to efficiently amplify the overexpressed transcript. Threshold cycles are indicated and error bars indicate standard deviation of values obtained based on two independent cDNA syntheses (technical replicates). **(b) **Detection of mature 21-bp Ath-miR859 and Ath-miR774 by Northern blot analysis in two representative independent transgenic lines (selected from (a)) overexpressing the Ath-*MIR859-774 *pri-*MIRNA*. Total RNAs (10 μg/lane) from each sample were blotted and probed with ^32^P-labeled standard DNA complementary to *U6 *RNA (loading control) or oligonucleotides complementary to mature miRNA sequences. **(c) **Real-time RT-PCR analysis of the relative accumulation of selected Ath-*MIR859-774 *validated targets (Ath-miR859 target TAIR:At3g49510 and Ath-miR774 target TAIR:At3g19890) in the two transgenic lines used in (b). The histogram represents amounts of specific PCR amplification products (verified by sequencing and dissociation curve analyses) normalized to reference genes [72] defined using Genorm software [73] (see Materials and methods). Values of target expression in the control line (Col-0) are set to 1. Error bars indicate the standard deviation of values obtained based on three independent cDNA syntheses (technical replicates).

To determine the functionality of a non-homologous polycistronic pri-*MIRNA in planta*, the Ath-*MIR859-774 *locus was selected. Expression of several Ath-*MIR859-774 *predicted targets encoding F-box proteins was analyzed in different organs (Figure 3c). Among validated targets, the Ath-miR774 target TAIR:At3g19890 and the Ath-miR859 target TAIR:At3g49510 exhibited detectable expression in roots that also express Ath-*MIR859-774 *pri-*MIRNA*. This indicates that both partners of this post-transcriptional regulation are present in this organ. We then overexpressed this precursor to analyze the transcriptional regulation of these target genes (Figure 4). Independent lines accumulating the pri-*MIRNA *transcripts at high levels in wild-type (Col-0) *Arabidopsis *plants were obtained (Figure 4a). Northern blot analyses showed accumulation of both mature approximately 21-bp miRNAs corresponding to Ath-miR859 and Ath-miR774 in comparison to control plants (expressing an empty vector; Figure 4b). In addition, significant down-regulation of both the Ath-miR859 target TAIR:At3g49510 and the Ath-miR774 target TAIR:At3g19890 was observed in these transgenic lines (Figure 4c). These results indicate that both miRNAs from the Ath-*MIR859-774 *polycistronic pri-*MIRNA *can be processed and simultaneously co-regulate the expression of their respective predicted targets.

**Figure 5:**
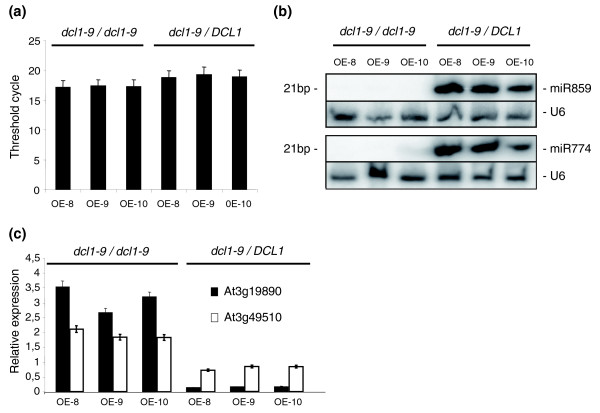
**Ath-*MIR859-774 *polycistronic pri-*MIRNA *is processed by a DCL1-dependent pathway**. **(a) **Overexpression of Ath-*MIR859-774 *pri-*MIRNA *in *dcl1.9 *mutant and in the corresponding *DCL1.9/dcl1.9 *heterozygous siblings (three representative OverExpressing (OE) transgenic lines) is shown. Real-time RT-PCR analysis was done on equal amounts of total RNAs. A green fluorescent protein encoding transcript present downstream of the overexpressed pri-*MIRNA *was used to efficiently detect the overexpressed transcript. Threshold cycles are indicated and error bars indicate standard deviation of values obtained based on two independent cDNA syntheses (technical replicates). **(b) **Detection of mature 21-bp Ath-miR859 and Ath-miR774 by Northern blot analysis in the three independent transgenic lines (shown in (a)) over-expressing Ath-*MIR859-774 *pri-*MIRNA *in *dcl1.9 *and in the *DCL1.9/dcl1.9 *heterozygous siblings. Total RNAs (10 μg/lane) from each sample were blotted and probed with ^32^P-labeled standard DNA complementary to *U6 *RNA (loading control) or modified oligonucleotides complementary to mature miRNA sequences. **(c) **Real-time RT-PCR analysis of the relative accumulation of selected Ath-*MIR859-774 *validated targets (Ath-miR859 target TAIR:At3g49510 and Ath-miR774 target TAIR:At3g19890) in the three transgenic lines (used in (a, b) overexpressing the corresponding pri-*MIRNA *in *dcl1.9 *and in the related *DCL1.9/dcl1.9 *heterozygous background. The histogram represents amounts of specific PCR amplification products (verified by sequencing and dissociation curve analyses) normalized to reference genes [72] defined using Genorm software [73] (see Materials and methods). Error bars indicate the standard deviation of values obtained based on three independent cDNA syntheses (technical replicates).

To determine whether the maturation of this atypical polycistronic *MIRNA *precursor depends on DCL1, which processes canonical single *MIRNA *precursors, the Ath-*MIR859-774 *overexpression construct was introduced into a *dcl1-9 *mutant. This weak DCL1 allele affects miRNA processing but is more viable than the embryonic lethal *dcl1 *null alleles [51]. Several independent lines overexpressing Ath-*MIR859-774 *did not accumulate Ath-miR859 and Ath-miR774 in aerial tissues of the homozygous *dcl1-9 *background (*dcl1-9/dcl1-9*), in contrast to the heterozygous siblings (*dcl1-9*/DCL1); Figure 5a, b). This indicates that the processing of polycistronic *MIRNA *precursors such as pri-*MIR859-774 *requires DCL1. Furthermore, down-regulation of the Ath-miR859 and Ath-miR774 targets was abolished in these *dcl1-9*/*dcl1-9 *transgenic lines (Figure 5c).

## Discussion

A comparative genomic analysis of miRNA clustering in three model plants (a monocot, rice, a herbaceous dicot, *Arabidopsis*, and a dicot tree, poplar) led us to identify a novel class of polycistronic *MIRNA *precursors encoding miRNAs with unrelated sequences. These non-homologous miRNA clusters target transcripts encoding proteins of the same family, suggesting that this unusual genomic organization may allow co-regulation of different but related targets.

Most miRNA clusters encode several copies of conserved miRNAs from the same family, that is, miR166, miR169, or miR395. Previous analyses of miR395 clusters in rice and *M. truncatula*, as well as a miR156 cluster in rice, maize, sugarcane, sorghum and even a dicot (*Ipomea nil*), have suggested conservation of homologous miRNA clusters in various plant genomes [16,29,30]. Our analysis revealed certain homologous miRNA clusters at syntenic genomic positions, implying a common evolutionary origin across monocot and dicot lineages. Specific miRNA families seem positively selected for expansion and clustering in several genomes. For cultivated species, it has been proposed that this spreading may contribute to advantageous agricultural traits [29,30]. In addition, homologous miRNAs or cluster duplication may lead to the emergence of new spatio-temporal expression patterns through the accommodation of alternative promoter regions [29,52,53].

A combination of tandem duplication of miRNAs as well as segmental duplications of whole clusters has been proposed to explain such genomic organization [29,52]. In animal genomes, miRNAs encoded in the miR17 cluster arose through a complex duplication and loss of individual members as well as duplications of entire clusters [28]. In plant genomes, miR156, miR160, miR162, miR167, miR169, miR171 and miR395 families experienced large expansions via tandem or segmental duplication events and loss of family members ([29,30,52] and this study). This is in agreement with the detection of two to three miRNAs in most (that is, 70 to 80%) of the clusters in our analysis, similar to protein coding gene clusters [52]. These duplication events may therefore represent a major evolutionary route for birth and death of miRNAs in plants.

Folding of putative transcripts derived from homologous miRNA clusters revealed additional hairpins in the rice Osa-*MIR395h-l *and Osa-*MIR395m-s *clusters, which were not annotated in miRBase. In animal genomes, systematic folding of genomic regions encoding miRNA clusters has helped to identify additional miRNAs [18]. A recent analysis of rice miRNA clusters has revealed a different genomic organization of upstream sequences corresponding to their promoters [53]. Osa-*MIR156b-c*, Osa-*MIR166k-h*, Osa-*MIR169n-o*, Osa-*MIR172b-806a*, Osa-*MIR395a-g*, Osa-*MIR395h-l*, and Osa-*MIR395m-s *clusters may contain only one promoter and be transcribed as polycistronic units. Interestingly, we found that the Osa-*MIR395t-w *cluster was specific to the rice genome. This cluster has previously been reported as having no predicted promoter [53]; a transposable element identified in its vicinity [29] may be associated with the recent evolution of this *MIRNA *cluster [6,54].

Our results indicate that short range (<1 kb) clustering of 'same DNA strand' miRNAs are highly suggestive of co-transcription as reported in animal genomes [18]. Accidental formation of hairpins followed by loss of miRNAs subsequent to duplication was indeed proposed as a general mechanism for the origin of polycistronic *MIRNA *transcripts in animals [28]. Although the clustered miRNAs characterized were always encoded in independent hairpins, a stem-loop encoding the rice miR159 was recently shown to produce additional approximately 21- to 24-nucleotide small RNAs from the 21 bp next to the miR159 sequence [33]. This unusual case is reminiscent of sequential DCL1-dependent processing of the *Arabidopsis *miR163, and of DCL4-dependent processing of tasiRNAs (trans-acting siRNAs) or young *Arabidopsis *miRNAs, which may correspond to 'proto-miRNAs' [6,55,56]. Our results show that maturation of the Ath-*MIR859-774 *polycistronic cluster is mediated by DCL1, but we cannot exclude that other (DCL) enzymatic complexes may contribute to the processing of polycistronic *MIRNA *precursors.

In each of the three plant genomes, we identified several clusters encoding distinct miRNAs, in addition to clusters containing homologous miRNAs. Their low abundance in plant genomes contrasts with animal genomes, where miRNA clusters frequently encode miRNAs from different, although evolutionarily related, families, for example, the miR17 gene cluster [25,28]. These clustered non-homologous *MIRNA *genes are proposed to simultaneously regulate multiple functionally related genes in animals. Indeed, a recent study has demonstrated that two human miRNA clusters regulate various cyclin dependent kinase inhibitors, leading to a coordinated regulation of cell cycle progression [27]. In contrast to animals where hundreds of translational targets are frequently predicted for a single miRNA, plant miRNAs target few transcripts, usually showing an extensive homology with the miRNA leading to its cleavage [42] (Table 2; Additional data file 4). Although the recent identification of translational regulation in plants may affect this view, all known translationally regulated targets presently contain binding sites highly homologous to miRNAs [5]. Interestingly, we show that all predicted targets of the different non-homologous miRNAs present in a single cluster always corresponded to proteins of the same family.

Ath-*MIR859-774 *and a representative target of each miRNA were mainly expressed in the roots. However, anti-correlation between the *MIRNA *precursor and target transcript levels was not identified in the different organs tested. This could be due to the fact that several plant miRNAs quantitatively regulate gene expression and a low level of variation in a specific organ could not be detected [6]. Indeed, both miRNA and targets were expressed at low levels in each organ tested (Figure 3; Figure S4 in Additional data file 3). Additionally, spatial expression domains of the miRNAs and their targets may vary in the different cell types constituting an organ, resulting in non-significant differences at the whole organ level or even positive correlations ([6,57] and references therein). Furthermore, post-translational regulations may be superimposed upon post-transcriptional regulations, as in the case of another recently evolved plant miRNA, miR834, initially suspected to be inactive [5]. In the latter case, the absence or near absence of transcriptional anti-correlation between miRNA and target transcripts suggests that post-translational regulation is predominant over mRNA regulation.

Ectopic expression of the Ath-*MIR859-774 *pri-*MIRNA *led to the simultaneous down-regulation of distinct F-box transcripts, which are likely to be independently regulated by each miRNA. F-box proteins co-regulated by Ath-*MIR859-774 *may participate in specific pathways involving proteasome-dependent degradation of signaling components [58]. Ptc-*MIR1446-477 *loci are predicted to target a DELLA-like transcription factor similar to the *Arabidopsis *RGL1/RGL2 proteins involved in gibberellin control of seed germination and floral development [45], and shoot and root development in poplar [59]. The Ath-*MIR397b-857 *targets transcripts encoding laccase copper proteins associated with lignin synthesis, metal nutrition and response to abiotic stresses [50,60]. Among the four laccase encoding transcripts targeted by these miRNAs, the knock-out mutant of TAIR:At2g29130 (AtLAC2) shows slightly reduced root elongation under osmotic stress. Finally, miRNAs derived from the Ath-*MIR842-846 *loci target transcripts encoding related JR/MBP, while Ptc-*MIR482-1448 *miRNAs target transcripts encoding disease resistance proteins. Both pathways may affect pathogen defense responses [44,61]. Co-transcription of similar or identical miRNAs has been proposed to have a dosage effect on target expression [29]. Co-expression of different miRNAs may serve to increase the efficiency of the regulatory process. Whereas different miRNAs have been shown to bind a single mRNA target in animal systems to cooperatively control its expression [19,62], only three Ath-*MIR859-774 *targets were predicted to be recognized by both miRNAs. This result might be biased due to the restrictive criteria used in plants to predict targets, in contrast to animal genomes [42].

*MIRNA *genes are proposed to originate from the duplication of a target gene [6,15,56,63]. In the case of polycistronic non-homologous *MIRNA *precursors, we could hypothesize that the duplication of a single target locus may have led to the selection of two divergent 'proto-miRNA' regions targeting other members of the family. An alternative is the duplication of an overlapping region between two clustered target genes, leading to the selection of miRNAs that target both clustered ancestral genes. Indeed, predicted targets of tandem polycistronic non-homologous *MIRNA *precursors are often themselves clustered (Additional data file 4), notably the F-box proteins targeted by Ath-*MIR859-774 *and the laccases targeted by Ath-*MIR397b-857 *(37 clustered F-box proteins and 4 clustered laccases).

Our results show that plant genomes generally contain less clustered or polycistronic miRNAs than animal genomes. Indeed, approximately 20% of total plant miRNAs are clustered, whereas in animals they represent approximately 50% using a similar criterion (that is, cluster size up to 10 kb) [18]. In animals, the Drosha complex specifically catalyzes maturation of long pri-*MIRNA*s, including the numerous polycistronic clusters, into approximately 70 nucleotide pre-*MIRNA*s hairpins [6]. In plants, however, a Drosha-like enzyme is lacking. We have shown that the processing of at least one *Arabidopsis *polycistronic *MIRNA *is DCL1-dependent, similar to most non-polycistronic *MIRNA *precursors. We can speculate that the occurrence of a single step maturation process of polycistronic precursors in plants may not be functionally equivalent to the two-step process existing in animals.

## Conclusions

In contrast to plants, clusters of miRNAs are frequently present in animal genomes. Our comparative genomic analysis in three model plants (rice, poplar and *Arabidopsis*), however, has demonstrated the presence of several clusters containing two to eight miRNA species. Certain ancestral miRNA clusters appeared before the divergence of monocot and dicot lineages, and showed differential expansions in plants. Specific miRNA clusters (such as those coding for miR395, miR169 and miR166) are highly conserved. Interestingly, other clusters comprise functional miRNAs with unrelated sequences (non-homologous miRNAs) and are expressed as single transcriptional units, defining a novel class of plant pri-*MIRNA *genes. These polycistronic non-homologous miRNAs regulate related target genes and are processed by a DCL1-dependent pathway. This mechanism paves the way for using polycistronic *MIRNA *precursors as a new molecular tool in plants to simultaneously express artificial miRNAs [64] that control the expression of different genes.

## Materials and methods

### Plant genotypes and growth conditions

The wild-type Columbia (Col-0) ecotype of *A. thaliana *was used, as well as a *dcl1-9 *mutant backcrossed five times to Col-0 [51,56]. All plants were grown in long day conditions (16-h light/8-h dark photoperiod) at 23°C. Inflorescences, stems and cauline leaves, or rosette leaves were collected from 3-week-old greenhouse-grown plants. Roots were collected from seedlings grown 3 weeks *in vitro *on 1/2 Murashige and Skoog (MS) medium (Sigma, Lyon, France) supplemented with 1% sucrose (Sigma, Lyon, France).

### Bioinformatic analysis

*Arabidopsis*, poplar and rice miRNA sequences (mature and precursor) were downloaded from the microRNA Registry version 13.0 [65]. miRNA coordinates, chromosome locations and DNA strand orientation were retrieved from the microRNA Registry.

*MIRNA *genes were sorted by their chromosome locations and coordinates to identify miRNA clusters. The distance between two consecutive *MIRNA *loci was calculated by subtracting the start coordinates of the downstream pre-*MIRNA *(that is, hairpin) to the end coordinates of the upstream pre-*MIRNA*. *MIRNA *loci located within a distance of less than 1, 3 or 10 kb were considered to define the best candidates for polycistronic clusters and clusters with stringent or non-stringent criteria, respectively. The DNA strand containing the miRNA sequence was considered in these analyses.

Secondary structures were predicted using the mfold program [66] with default parameters [67] and a window size between 1 and 2 kb depending on *MIRNA *precursors.

### Conservation analysis of miRNA clusters between plant genomes

Conservation between selected clustered miRNAs in *Arabidopsis*, poplar, sorghum and rice genomes as well as determination of candidate orthologous regions were determined using Genome VISTA [36,37]. Query sequence (1 to 1.5 kb depending on clusters) was anchored on the reference genome by local alignment matches and then globally aligned to candidate regions in different selected genomes based on the AVID program [68,69]. Alignments were then displayed with the VISTA graphic server [70]. Identified syntenic regions were manually inspected to identify and annotate orthologous miRNA clusters.

### Northern blot analysis of small RNA expression

Tissues were frozen in liquid nitrogen, ground to a fine powder with a mortar and pestle, and then homogenized in TRI-Reagent^® ^(Sigma, Lyon, France) supplemented with β-mercaptoethanol. Total RNAs were prepared according to the manufacturer's instructions (Sigma, Lyon, France) with additional steps: samples were extracted with one volume of Tris/HCl-buffered phenol/chloroform (Sigma, Lyon, France), then with two volumes of chloroform, and finally RNAs were precipitated with three volumes of ice-cold 100% ethanol and one-tenth volume of 3 M sodium acetate (pH 6) in diethylenepyrocarbonate (DEPC)-treated water. Northern blot analysis of low molecular weight RNAs (10 μg of total RNAs per lane) was carried out on denaturing 15% polyacrylamide (19:1) gels cast in 7 M urea/Tris borate EDTA (TBE) buffer, followed by blotting onto a nylon hybridization membrane (Hybond-NX^®^, Amersham/Pharmacia, Les Ulis, France) pre-wetted in distilled water. An EDC (1-ethyl-3- [3-dimethylaminopropyl]carbodiimide hydrochloride)-mediated cross-linking step was then performed as described [71]. Blots were hybridized with gamma-ATP ^32^P end-labeled oligonucleotides (20 pmoles) complementary to miRNAs, and at the same time with an end-labelled oligonucleotide U6 RNA probe as loading control.

### Analysis of gene expression by RT-PCR

Total RNAs were extracted using the total RNA Isolation kit (Macherey-Nagel, Düren Germany). cDNA was synthesized by reverse transcription of 1.5 μg of total RNAs using the SuperScript II Reverse Transcriptase (Invitrogen, Paisley, UK) and (T)16 A/G/C oligonucleotides. Primer pairs used for RT-PCR are listed in Additional data file 5. Specificity of amplification was checked by cloning and sequencing of PCR amplicons, and ESTs corresponding to *Arabidopsis *non-homologous pri-*MIRNA*s were submitted to GenBank ([NCBI:GU125419] for Ath-*MIR859-774*; [NCBI:GU125420] for Ath-*MIR850-863*; [NCBI:GU125421] for Ath-*MIR397B-857*; [NCBI:GU125422] for Ath-*MIR851-771*). For Ath-*MIR842-846 *loci, no amplification was obtained despite testing eight different primer combinations, even on genomic DNA (data not shown). A control without reverse transcriptase was systematically included.

Real-time RT-PCR was performed on an Eppendorf Mastercycler^® ^realplex real-time PCR system (Eppendorf, Hamburg, Germany) using FastStart Universal SYBR Green Master Mix (Rox) from Roche Applied Science (Meylan, France). Technical triplicates were done for each datapoint, and two independent biological replicates (per condition and/or transgenic line) were assayed. Normalization was done with averaged reference genes TAIR:At1g13320, TAIR:At4g26410, and TAIR:At5g15710 [72], which were systematically validated under our experimental conditions using Genorm software [73].

### Cloning and transgenic plants

Firstly, pri-*MIRNA *Ath-*MIR859-774 *was amplified by RT-PCR from seedling cDNA and cloned into pCR8^®^/GW/TOPO^® ^TA Cloning^® ^vector (Invitrogen, Paisley, UK). The construct was then transferred to the destination vector pEarlyGate103 [74] using the LR recombination kit (Invitrogen, Paisley, UK). These constructions (based on the 35S-CaMV promoter) were used to transform *A. thaliana *plants by floral dipping [75]. Transgenic plants were selected in T1 generation by spraying seedlings with Basta^® ^solution (120 mg/L glufosinate ammonium; Bayer CropScience, Monheim am Rhein, Germany) successively at 12, 14, and 16 days after germination. Basta-resistant plantlets were then tested for transgene expression by real time RT-PCR as described above. Since amplification across the successive hairpin regions of the Ath-*MIR859-774 *pri-*MIRNA *was not efficient and quantitative enough for real time RT-PCR analyses, a *GFP *mRNA present 3' of the pEarlyGate103 vector cloning site, for which efficient and specific primers were available (Additional data file 5), was used as a 3' tag to analyze transgene expression.

## Abbreviations

ASRP: *Arabidopsis *Small RNA Project; Ath: *Arabidopsis thaliana*; DCL1: Dicer-like enzyme 1; EST: expressed sequence tag; JR/MBP: Jacalin repeat/Myrosinase binding protein; miRNA: microRNA; Osa: *Oryza sativ*a; pre-*MIRNA*: miRNA precursor; pri-*MIRNA*: miRNA primary transcript; Ptc: *Populus trichocarpa*; RGL: Repressor of gibberellic acid requiring (GA1)-LIKE; RISC: RNA-induced silencing complex; TAIR: The *Arabidopsis *Information Resource.

## Authors' contributions

FM carried out the molecular genetic studies. AB conceived the study and carried out the bioinformatic analyses. MC drafted the manuscript and participated in its coordination. FF conceived the study, designed experiments and wrote the manuscript. All authors read and approved the final manuscript.

## Additional data files

The following additional data are available with the online version of this paper: a table listing the conserved and non-conserved miRNAs in *Arabidopsis*, rice and poplar genomes (Additional data file 1); a table providing a detailed list of clustered miRNAs in *Arabidopsis*, rice and poplar genomes (Additional data file 2); a PDF including Figures S1 to S4 (Additional data file 3); a table providing a detailed list of all targets predicted for *Arabidopsis *non-homologous polycistronic miRNA clusters (Additional data file 4); a table listing primers used in this study (Additional data file 5).

## Supplementary Material

Additional data file 1 Conserved and non-conserved miRNAs in *Arabidopsis*, rice and poplar genomes. Click here for file

Additional data file 2 Clustered miRNAs in *Arabidopsis*, rice and poplar genomes. Click here for file

Additional data file 3 Figure S1: secondary structures of the rice osa-*MIR395a-g *and osa-*MIR395h-l,y *putative polycistronic homologous miRNA clusters. Figure S2: secondary structures of poplar and rice putative polycistronic non-homologous miRNA clusters. Figure S3: secondary structures of four *Arabidopsis *non-homologous polycistronic miRNA clusters: Ath-*MIR397b-857*, ath-*MIR842-846*, ath-*MIR850-863*, and ath-*MIR851-771*. Figure S4: small RNA hits in *Arabidopsis *polycistronic non-homologous miRNA clusters based on the 'Genome View' browser in the ASRP database. Click here for file

Additional data file 4 Targets predicted for *Arabidopsis *non-homologous polycistronic miRNA clusters. Click here for file

Additional data file 5 Primers used in this study. Click here for file

## References

[B1] Bartel DP MicroRNAs: genomics, biogenesis, mechanism, and function. Cell 2004 116 281 297 10.1016/S0092-8674(04)00045-5 14744438

[B2] Mallory AC Vaucheret H Functions of microRNAs and related small RNAs in plants. Nat Genet 2006 38 31 36 10.1038/ng1791 16736022

[B3] Jones-Rhoades MW Bartel DP Computational identification of plant microRNAs and their targets, including a stress-induced miRNA. Mol Cell 2004 14 787 799 10.1016/j.molcel.2004.05.027 15200956

[B4] Xiao C Rajewsky K MicroRNA control in the immune system: basic principles. Cell 2009 136 26 36 10.1016/j.cell.2008.12.027 19135886

[B5] Brodersen P Sakvarelidze-Achard L Bruun-Rasmussen M Dunoyer P Yamamoto YY Sieburth L Voinnet O Widespread translational inhibition by plant miRNAs and siRNAs. Science 2008 320 1185 1190 10.1126/science.1159151 18483398

[B6] Voinnet O Origin, biogenesis, and activity of plant microRNAs. Cell 2009 136 669 687 10.1016/j.cell.2009.01.046 19239888

[B7] Chapman EJ Carrington JC Specialization and evolution of endogenous small RNA pathways. Nat Rev Genet 2007 8 884 896 10.1038/nrg2179 17943195

[B8] Mallory AC Elmayan T Vaucheret H MicroRNA maturation and action - the expanding roles of ARGONAUTEs. Curr Opin Plant Biol 2008 11 560 566 10.1016/j.pbi.2008.06.008 18691933

[B9] Chen X MicroRNA metabolism in plants. Curr Top Microbiol Immunol 2008 320 117 136 full_text 18268842 10.1007/978-3-540-75157-1_6 PMC2570777

[B10] Ramachandran V Chen X Small RNA metabolism in *Arabidopsis*. Trends Plant Sci 2008 13 368 374 10.1016/j.tplants.2008.03.008 18501663 PMC2569976

[B11] Kim VN Han J Siomi MC Biogenesis of small RNAs in animals. Nat Rev Mol Cell Biol 2009 10 126 139 10.1038/nrm2632 19165215

[B12] Bollman KM Aukerman MJ Park MY Hunter C Berardini TZ Poethig RS HASTY, the *Arabidopsis *ortholog of exportin 5/MSN5, regulates phase change and morphogenesis. Development 2003 130 1493 1504 10.1242/dev.00362 12620976

[B13] Park MY Wu G Gonzalez-Sulser A Vaucheret H Poethig RS Nuclear processing and export of microRNAs in *Arabidopsis*. Proc Natl Acad Sci USA 2005 102 3691 3696 10.1073/pnas.0405570102 15738428 PMC553294

[B14] Griffiths-Jones S Saini HK van Dongen S Enright AJ miRBase: tools for microRNA genomics. Nucleic Acids Res 2009 36 D154 D158 10.1093/nar/gkm952 PMC2238936 17991681

[B15] Fahlgren N Howell MD Kasschau KD Chapman EJ Sullivan CM Cumbie JS Givan SA Law TF Grant SR Dangl JL Carrington JC High-throughput sequencing of *Arabidopsis *microRNAs: evidence for frequent birth and death of *MIRNA *genes. PLoS ONE 2007 2 e219 10.1371/journal.pone.0000219 17299599 PMC1790633

[B16] Sunkar R Jagadeeswaran G *In silico *identification of conserved microRNAs in large number of diverse plant species. BMC Plant Biol 2008 8 37 10.1186/1471-2229-8-37 18416839 PMC2358906

[B17] Rodriguez A Griffiths-Jones S Ashurst JL Bradley A Identification of mammalian microRNA host genes and transcription units. Genome Res 2004 14 1902 1910 10.1101/gr.2722704 15364901 PMC524413

[B18] Altuvia Y Landgraf P Lithwick G Elefant N Pfeffer S Aravin A Brownstein MJ Tuschl T Margalit H Clustering and conservation patterns of human microRNAs. Nucleic Acids Res 2005 33 2697 2706 10.1093/nar/gki567 15891114 PMC1110742

[B19] Kim VN Nam JW Genomics of microRNA. Trends Genet 2006 22 165 173 10.1016/j.tig.2006.01.003 16446010

[B20] Lagos-Quintana M Rauhut R Lendeckel W Tuschl T Identification of novel genes coding for small expressed RNAs. Science 2001 294 853 858 10.1126/science.1064921 11679670

[B21] Lau NC Lim LP Weinstein EG Bartel DP An abundant class of tiny RNAs with probable regulatory roles in *Caenorhabditis elegans*. Science 2001 294 858 862 10.1126/science.1065062 11679671

[B22] Mourelatos Z Dostie J Paushkin S Sharma A Charroux B Abel L Rappsilber J Mann M Dreyfuss G miRNPs: a novel class of ribonucleoproteins containing numerous microRNAs. Genes Dev 2002 16 720 728 10.1101/gad.974702 11914277 PMC155365

[B23] Aravin AA Lagos-Quintana M Yalcin A Zavolan M Marks D Snyder B Gaasterland T Meyer J Tuschl T The small RNA profile during *Drosophila melanogaster *development. Dev Cell 2003 5 337 350 10.1016/S1534-5807(03)00228-4 12919683

[B24] Lai EC Tomancak P Williams RW Rubin GM Computational identification of *Drosophila *microRNA genes. Genome Biol 2003 4 R42 10.1186/gb-2003-4-7-r42 12844358 PMC193629

[B25] Yu J Wang F Yang GH Wang FL Ma YN Du ZW Zhang JW Human microRNA clusters: genomic organization and expression profile in leukemia cell lines. Biochem Biophys Res Commun 2006 349 59 68 10.1016/j.bbrc.2006.07.207 16934749

[B26] Thatcher EJ Bond J Paydar I Patton JG Genomic organization of zebrafish microRNAs. BMC Genomics 2008 9 253 10.1186/1471-2164-9-253 18510755 PMC2427041

[B27] Kim YK Yu J Han TS Park SY Namkoong B Kim DH Hur K Yoo MW Lee HJ Yang HK Kim VN Functional links between clustered microRNAs: suppression of cell-cycle inhibitors by microRNA clusters in gastric cancer. Nucleic Acids Res 2009 37 1672 1681 10.1093/nar/gkp002 19153141 PMC2655672

[B28] Tanzer A Stadler PF Molecular evolution of a microRNA cluster. J Mol Biol 2004 339 327 335 10.1016/j.jmb.2004.03.065 15136036

[B29] Guddeti S Zhang DC Li AL Leseberg CH Kang H Li XG Zhai WX Johns MA Mao L Molecular evolution of the rice miR395 gene family. Cell Res 2005 15 631 638 10.1038/sj.cr.7290333 16117853

[B30] Wang S Zhu QH Guo X Gui Y Bao J Helliwell C Fan L Molecular evolution and selection of a gene encoding two tandem microRNAs in rice. FEBS Lett 2007 581 4789 4793 10.1016/j.febslet.2007.09.002 17884044

[B31] Chuck G Cigan AM Saeteurn K Hake S The heterochronic maize mutant *corngrass1 *results from overexpression of a tandem microRNA. Nat Genet 2007 39 544 549 10.1038/ng2001 17369828

[B32] Boualem A Laporte P Jovanovic M Laffont C Plet J Combier JP Niebel A Crespi M Frugier F MicroRNA166 controls root and nodule development in *Medicago truncatula*. Plant J 2008 54 876 887 10.1111/j.1365-313X.2008.03448.x 18298674

[B33] Lacombe S Nagasaki H Santi C Duval D Piegu B Bangratz M Breitler JC Guiderdoni E Brugidou C Hirsch J Cao X Brice C Panaud O Karlowski WM Sato Y Echeverria M Identification of precursor transcripts for 6 novel miRNAs expands the diversity on the genomic organisation and expression of miRNA genes in rice. BMC Plant Biol 2008 8 123 142 10.1186/1471-2229-8-123 19055717 PMC2607281

[B34] Lee Y Jeon K Lee JT Kim S Kim VN MicroRNA maturation: stepwise processing and subcellular localization. EMBO J 2002 21 4663 4670 10.1093/emboj/cdf476 12198168 PMC126204

[B35] Baskerville S Bartel DP Microarray profiling of microRNAs reveals frequent coexpression with neighboring miRNAs and host genes. RNA 2005 11 241 247 10.1261/rna.7240905 15701730 PMC1370713

[B36] VISTA Plot http://genome.lbl.gov/vista/index.shtml

[B37] Frazer KA Pachter L Poliakov A Rubin EM Dubchak I VISTA: computational tools for comparative genomics. Nucleic Acids Res 2004 32 W273 W279 10.1093/nar/gkh458 15215394 PMC441596

[B38] Gustafson AM Allen E Givan S Smith D Carrington JC Kasschau KD ASRP: the *Arabidopsis *Small RNA Project database. Nucleic Acids Res 2005 33 D637 D640 10.1093/nar/gki127 15608278 PMC540081

[B39] Backman TW Sullivan CM Cumbie JS Miller ZA Chapman EJ Fahlgren N Givan SA Carrington JC Kasschau KD Update of ASRP: the *Arabidopsis *Small RNA Project database. Nucleic Acids Res 2008 36 D982 D985 10.1093/nar/gkm997 17999994 PMC2238918

[B40] *Arabidopsis *Small RNA Project http://asrp.cgrb.oregonstate.edu/

[B41] Meyers BC Axtell MJ Bartel B Bartel DP Baulcombe D Bowman JL Cao X Carrington JC Chen X Green PJ Griffiths-Jones S Jacobsen SE Mallory AC Martienssen RA Poethig RS Qi Y Vaucheret H Voinnet O Watanabe Y Weigel D Zhu JK Criteria for annotation of plant MicroRNAs. Plant Cell 2008 20 3186 3190 10.1105/tpc.108.064311 19074682 PMC2630443

[B42] Brodersen P Voinnet O Revisiting the principles of microRNA target recognition and mode of action. Nat Rev Mol Cell Biol 2009 10 141 148 10.1038/nrm2619 19145236

[B43] Lu S Sun YH Shi R Clark C Li L Chiang VL Novel and mechanical stress-responsive MicroRNAs in *Populus trichocarpa *that are absent from *Arabidopsis*. Plant Cell 2005 17 2186 2203 10.1105/tpc.105.033456 15994906 PMC1182482

[B44] Lu S Sun YH Chiang VL Stress-responsive microRNAs in *Populus* Plant J 2008 55 131 151 10.1111/j.1365-313X.2008.03497.x 18363789

[B45] Tyler L Thomas SG Hu J Dill A Alonso JM Ecker JR Sun TP Della proteins and gibberellin-regulated seed germination and floral development in *Arabidopsis*. Plant Physiol 2004 135 1008 1019 10.1104/pp.104.039578 15173565 PMC514135

[B46] *Populus trichocarpa *v1.1 http://genome.jgi-psf.org/Poptr1_1/Poptr1_1.info.html

[B47] AtPARE Database http://mpss.udel.edu/at_pare/

[B48] Lu C Kulkarni K Souret FF MuthuValliappan R Tej SS Poethig RS Henderson IR Jacobsen SE Wang W Green PJ Meyers BC MicroRNAs and other small RNAs enriched in the *Arabidopsis *RNA-dependent RNA polymerase-2 mutant. Genome Res 2006 16 1276 1288 10.1101/gr.5530106 16954541 PMC1581437

[B49] Jones-Rhoades MW Bartel DP Computational identification of plant microRNAs and their targets, including a stress-induced miRNA. Mol Cell 2004 14 787 799 10.1016/j.molcel.2004.05.027 15200956

[B50] Abdel-Ghany SE Pilon M MicroRNA-mediated systemic down-regulation of copper protein expression in response to low copper availability in *Arabidopsis*. J Biol Chem 2008 283 15932 15945 10.1074/jbc.M801406200 18408011 PMC3259626

[B51] Schauer SE Jacobsen SE Meinke DW Ray A DICER-LIKE1: blind men and elephants in *Arabidopsis *development. Trends Plant Sci 2002 7 487 491 10.1016/S1360-1385(02)02355-5 12417148

[B52] Maher C Stein L Ware D Evolution of *Arabidopsis *microRNA families through duplication events. Genome Res 2006 16 510 519 10.1101/gr.4680506 16520461 PMC1457037

[B53] Cui X Xu SM Mu DS Yang ZM Genomic analysis of rice microRNA promoters and clusters. Gene 2009 431 61 66 10.1016/j.gene.2008.11.016 19073239

[B54] Piriyapongsa J Jordan IK Dual coding of siRNAs and miRNAs by plant transposable elements. RNA 2008 14 814 821 10.1261/rna.916708 18367716 PMC2327354

[B55] Vazquez F Vaucheret H Rajagopalan R Lepers C Gasciolli V Mallory AC Hilbert JL Bartel DP Crete P Endogenous trans-acting siRNAs regulate the accumulation of *Arabidopsis *mRNAs. Mol Cell 2004 16 69 79 10.1016/j.molcel.2004.09.028 15469823

[B56] Rajagopalan R Vaucheret H Trejo J Bartel DP A diverse and evolutionarily fluid set of microRNAs in *Arabidopsis thaliana*. Genes Dev 2006 20 3407 3425 10.1101/gad.1476406 17182867 PMC1698448

[B57] Kawashima CG Yoshimoto N Maruyama-Nakashita A Tsuchiya YN Saito K Takahashi H Dalmay T Sulphur starvation induces the expression of microRNA-395 and one of its target genes but in different cell types. Plant J 2009 57 313 321 10.1111/j.1365-313X.2008.03690.x 18801012

[B58] Lechner E Achard P Vansiri A Potuschak T Genschik P F-box proteins everywhere. Curr Opin Plant Biol 2006 9 631 638 10.1016/j.pbi.2006.09.003 17005440

[B59] Busov V Meilan R Pearce DW Rood SB Ma C Tschaplinski TJ Strauss SH Transgenic modification of gai or rgl1 causes dwarfing and alters gibberellins, root growth, and metabolite profiles in *Populus* Planta 2006 224 288 299 10.1007/s00425-005-0213-9 16404575

[B60] Cai X Davis EJ Ballif J Liang M Bushman E Haroldsen V Torabinejad J Wu Y Mutant identification and characterization of the laccase gene family in *Arabidopsis* J Exp Bot 2006 57 2563 2569 10.1093/jxb/erl022 16804053

[B61] Clay NK Adio AM Denoux C Jander G Ausubel FM Glucosinolate metabolites required for an *Arabidopsis *innate immune response. Science 2009 323 95 101 10.1126/science.1164627 19095898 PMC2630859

[B62] Lewis BP Shih IH Jones-Rhoades MW Bartel DP Burge CB Prediction of mammalian microRNA targets. Cell 2003 115 787 798 10.1016/S0092-8674(03)01018-3 14697198

[B63] Allen E Xie Z Gustafson AM Sung GH Spatafora JW Carrington JC Evolution of microRNA genes by inverted duplication of target gene sequences in *Arabidopsis thaliana*. Nat Genet 2004 36 1282 1290 10.1038/ng1478 15565108

[B64] Schwab R Ossowski S Riester M Warthmann N Weigel D Highly specific gene silencing by artificial microRNAs in *Arabidopsis* Plant Cell 2006 18 1121 1133 10.1105/tpc.105.039834 16531494 PMC1456875

[B65] microRNA Registry http://www.sanger.ac.uk/Software/Rfam/mirna

[B66] mfold Program http://mfold.bioinfo.rpi.edu/cgi-bin/rna-form1.cgi

[B67] Zuker M Mfold web server for nucleic acid folding and hybridization prediction. Nucleic Acids Res 2003 31 3406 3415 10.1093/nar/gkg595 12824337 PMC169194

[B68] Couronne O Poliakov A Bray N Ishkhanov T Ryaboy D Rubin E Pachter L Dubchak I Strategies and tools for whole-genome alignments. Genome Res 2003 13 73 80 10.1101/gr.762503 12529308 PMC430965

[B69] Bray N Dubchak I Pachter L AVID: A global alignment program. Genome Res 2003 13 97 102 10.1101/gr.789803 12529311 PMC430967

[B70] VISTA Graphic Server http://genome.lbl.gov/vista/index.shtml

[B71] Pall GS Codony-Servat C Byrne J Ritchie L Hamilton A Carbodiimide-mediated cross-linking of RNA to nylon membranes improves the detection of siRNA, miRNA and piRNA by northern blot. Nucleic Acids Res 2007 35 e60 10.1093/nar/gkm112 17405769 PMC1885651

[B72] Czechowski T Stitt M Altmann T Udvardi MK Scheible WR Genome-wide identification and testing of superior reference genes for transcript normalization in *Arabidopsis*. Plant Physiol 2005 139 5 17 10.1104/pp.105.063743 16166256 PMC1203353

[B73] Vandesompele J De Preter K Pattyn F Poppe B Van Roy N De Paepe A Speleman F Accurate normalization of real-time quantitative RT-PCR data by geometric averaging of multiple internal control genes. Genome Biol 2002 3 10.1186/gb-2002-3-7-research0034 12184808 PMC126239

[B74] pEarlyGate Vectors http://www.biology.wustl.edu/pikaard/Vectors%20homepage.html

[B75] Bechtold N Pelletier G In planta *Agrobacterium*-mediated transformation of adult *Arabidopsis thaliana *plants by vacuum infiltration. Methods Mol Biol 1998 82 259 266 9664431 10.1385/0-89603-391-0:259

