# Supplementary material for: Plant polycistronic precursors containing non-homologous microRNAs target transcripts encoding functionally related proteins
Source: Genome Biol. 2009 Dec 1;10(12):R136. doi: 10.1186/gb-2009-10-12-r136 (PMC2812943; doi:10.1186/gb-2009-10-12-r136)
Supplement: Additional data file 3 — Figure S1: secondary structures of the rice osa-MIR395a-g and osa-MIR395h-l,y putative polycistronic homologous miRNA clusters. Figure S2: secondary structures of poplar and rice putative polycistronic non-homologous miRNA clusters. Figure S3: secondary structures of four Arabidopsis non-homologous polycistronic miRNA clusters: Ath-MIR397b-857, ath-MIR842-846, ath-MIR850-863, and ath-MIR851-771. Figure S4: small RNA hits in Arabidopsis polycistronic non-homologous miRNA clusters based on the 'Genome View' browser in the ASRP database. [file gb-2009-10-12-r136-S3.PDF]

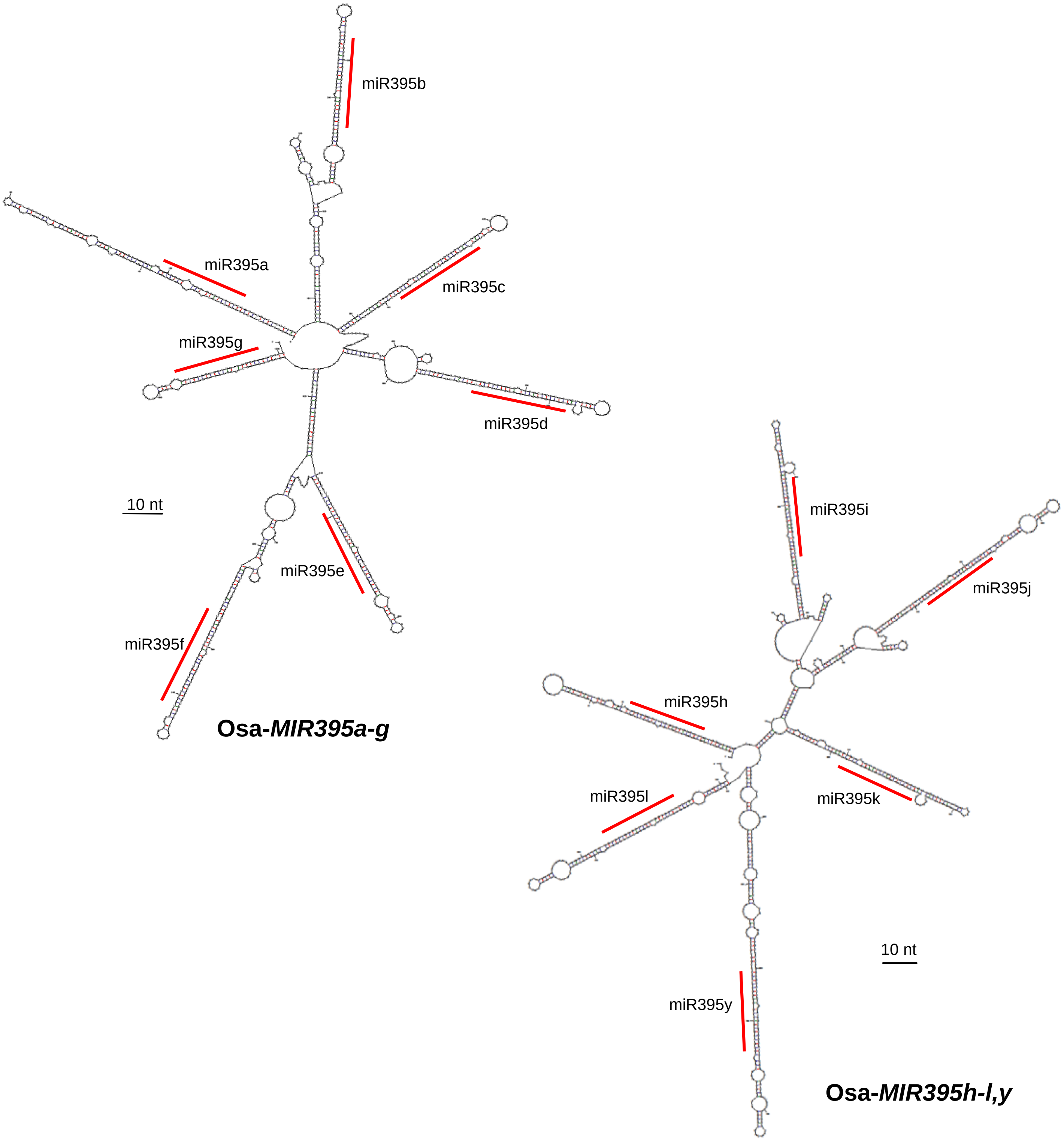

Figure S1. Secondary structures of the rice Osa-*MIR395a-g* and Osa-*MIR395h-l,y* putative polycistronic homologous miRNA clusters (in addition to the Osa-*MIR395m-s,x* cluster shown in Figure 2)
Mfold software [66, 67] was used to generate most probable secondary structures of clustered *MIRNA* loci. Mature miRNA sequences are indicated with a red line. In the case of the Osa-*MIR395h-l,y* cluster, a new MIR395 locus (Osa-*MIR395y*) was annotated as compared to data available in miRBase (version 13.0).
Osa: *Oryza sativa*; black bar= 10 nucleotides (nt).

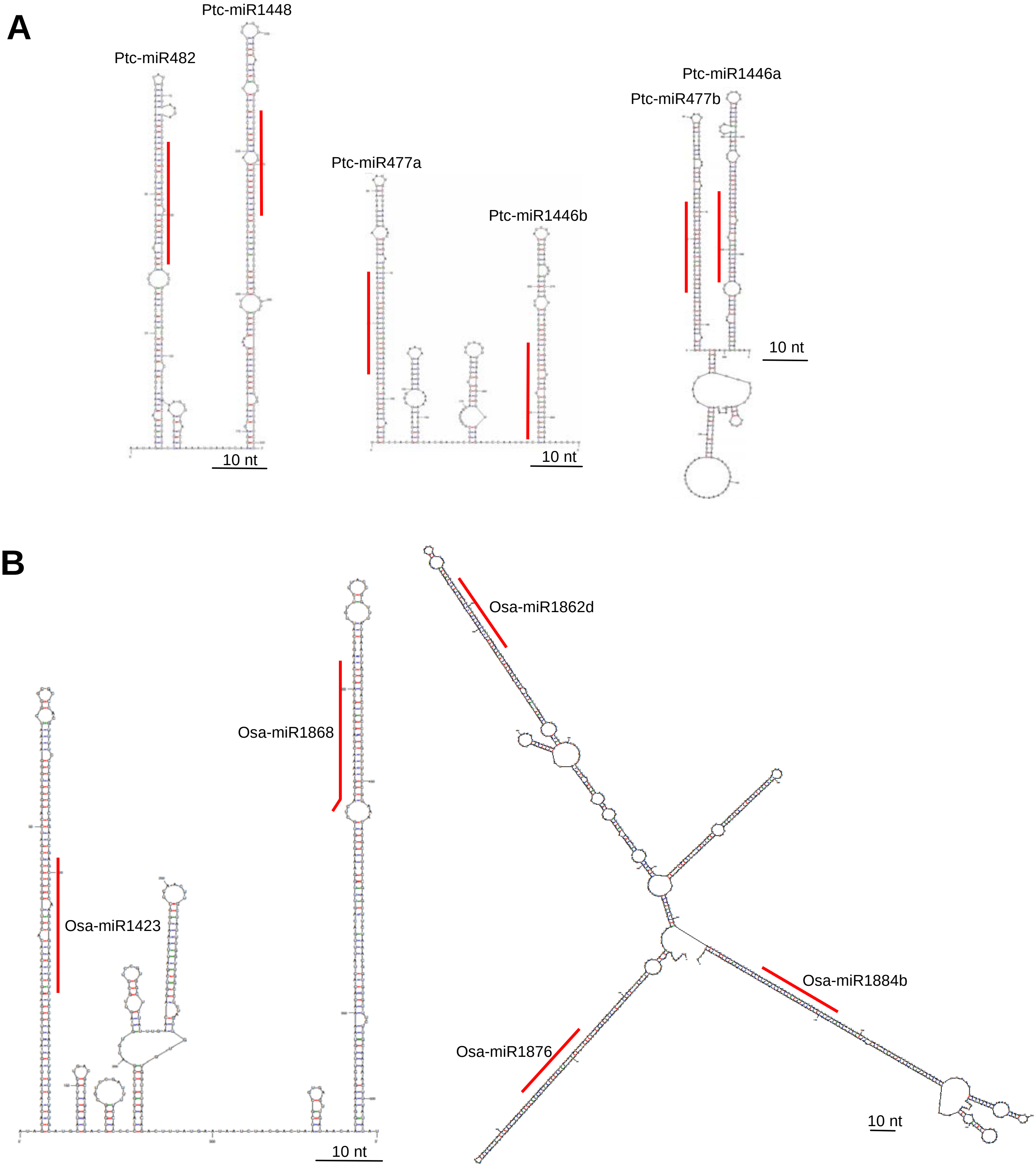

Figure S2. Secondary structures of poplar (A) and rice (B) putative polycistronic non-homologous miRNA clusters

Mfold software [66, 67] was used to generate most probable secondary structures of clustered *MIRNA* loci. Mature miRNA sequences are indicated with a red line.

Osa: *Oryza sativa*; Ptc: *Populus trichocarpa;* black bar= 10 nucleotides (nt).

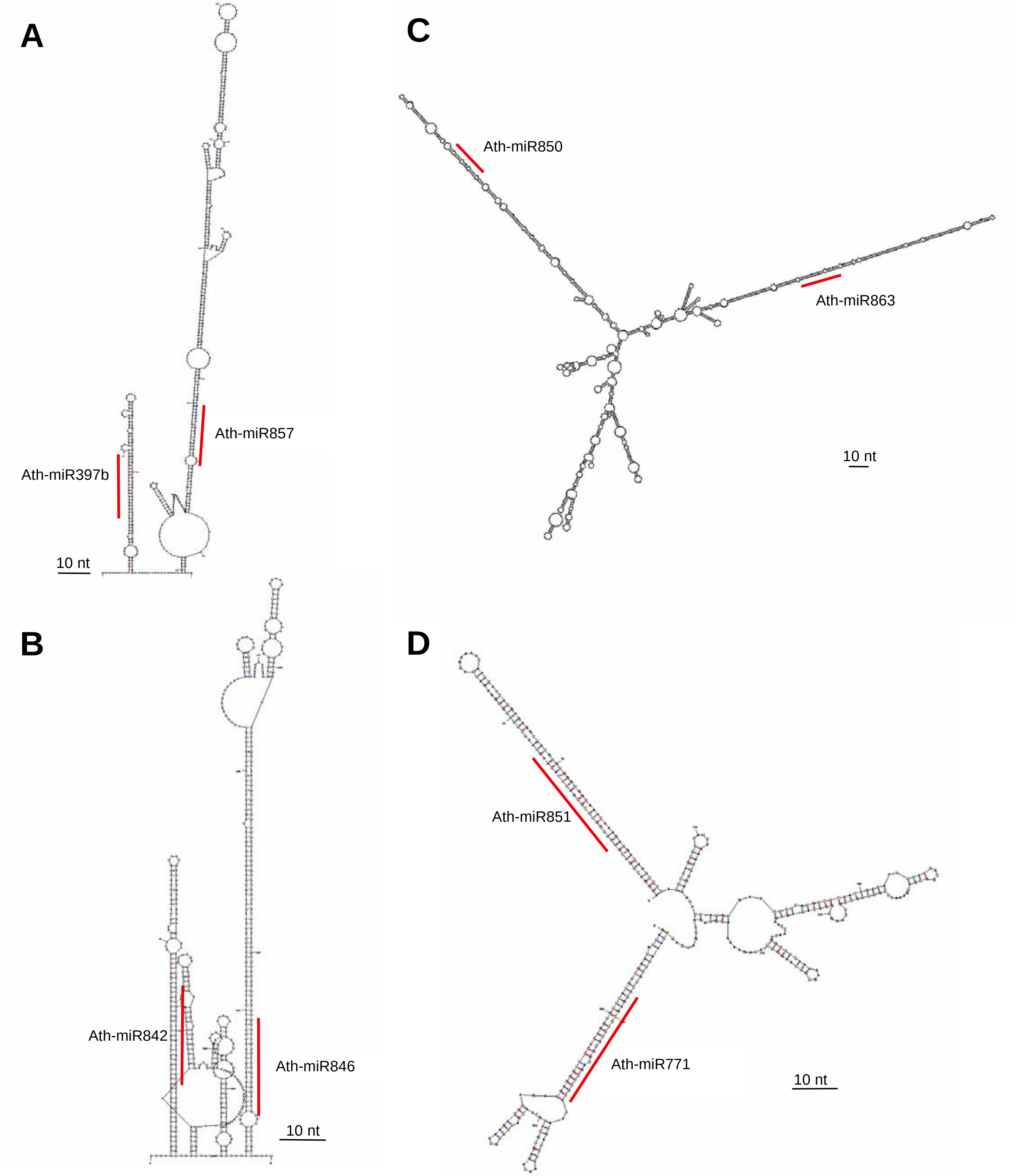

Figure S3. Secondary structures of four *Arabidopsis* non-homologous polycistronic miRNA clusters (in addition to the Ath-*MIR859-774* cluster shown in Figure 2): Ath-*MIR397b-857* (A), Ath-*MIR842-846* (B), Ath-*MIR850-863* (C), and Ath-*MIR851-771* (D)

Mfold software [66, 67] was used to generate most probable secondary structures of clustered *MIRNA* loci. Mature miRNA sequences are indicated with a red line.

Ath: *A. thaliana;* black bar= 10 nucleotides (nt).

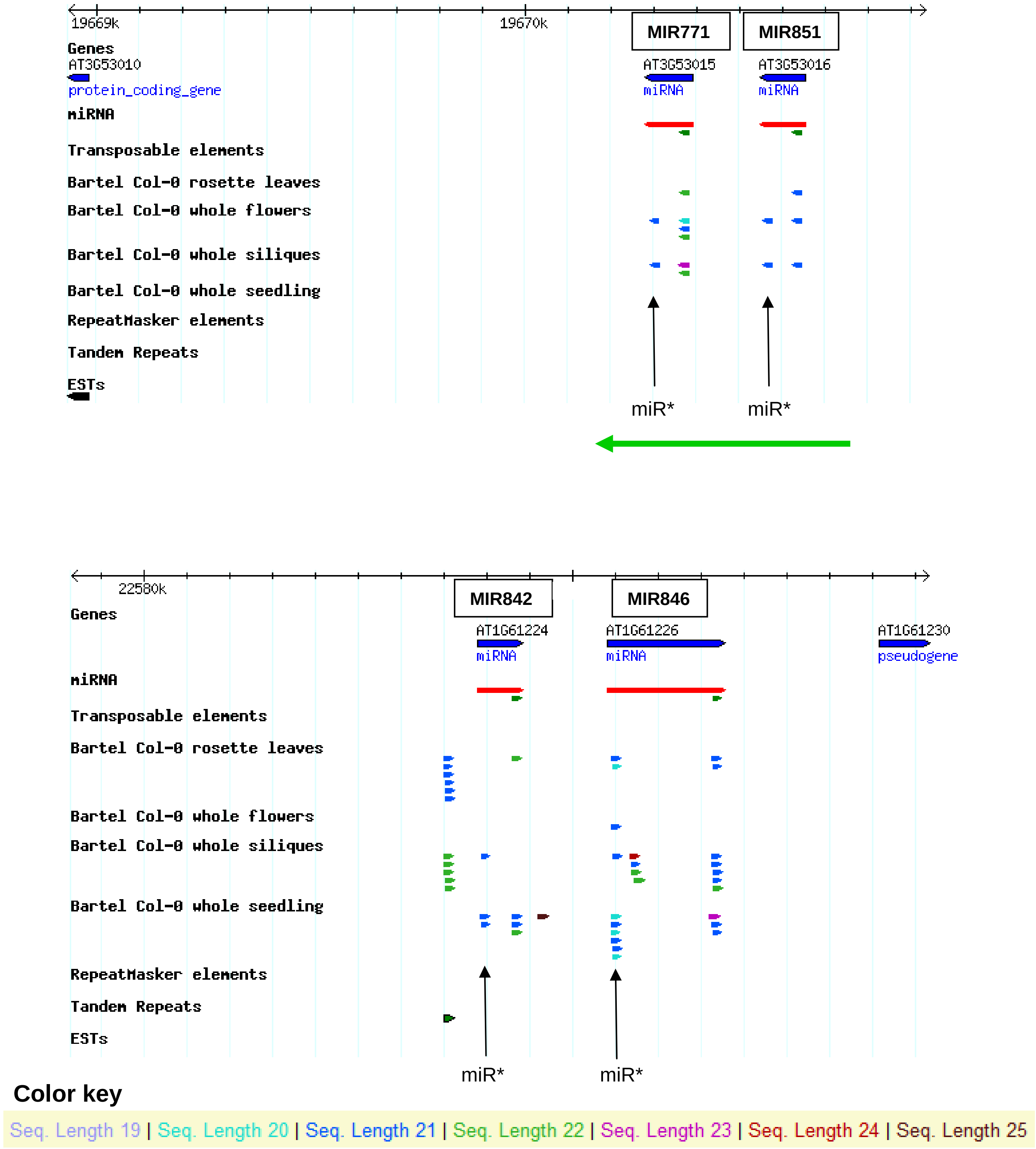

Figure S4. Small RNA hits in *Arabidopsis* polycistronic non-homologous miRNA clusters (based on the "Genome View" browser in ASRP database; http://asrp.cgrb.oregonstate.edu/).
Green arrows represent polycistronic ESTs, encompassing both predicted miRNA hairpins, which were amplified by RT-PCR in this study.

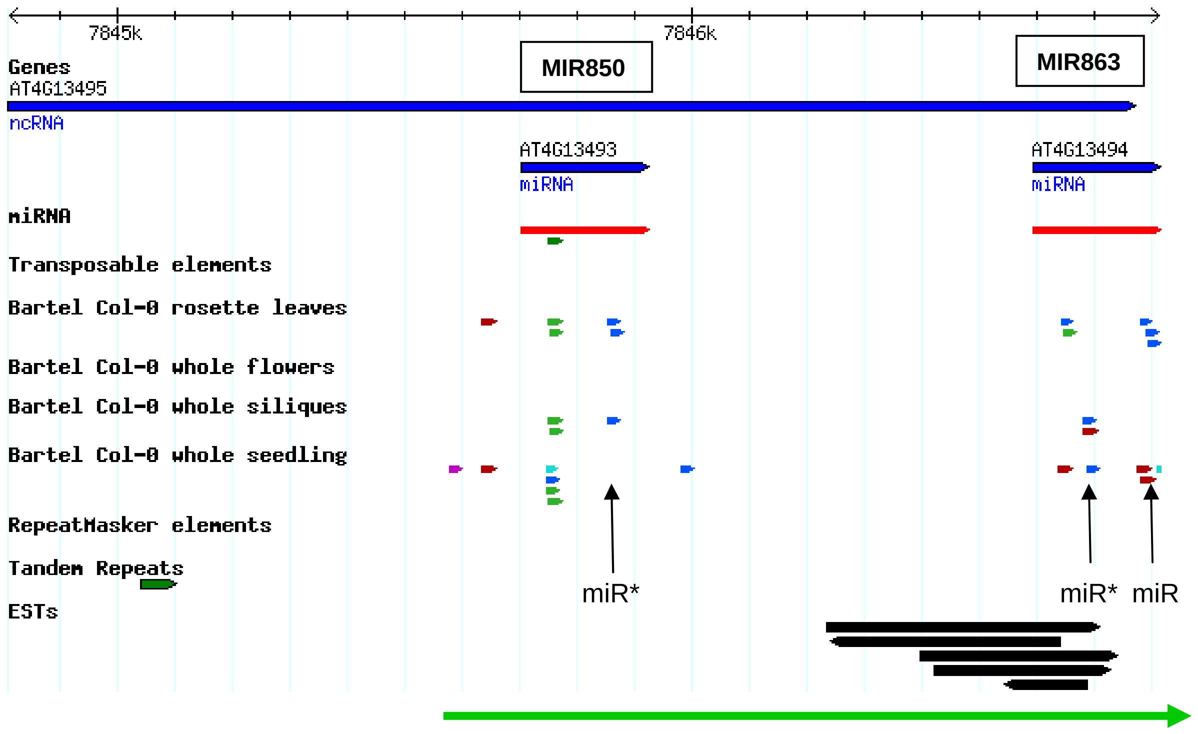

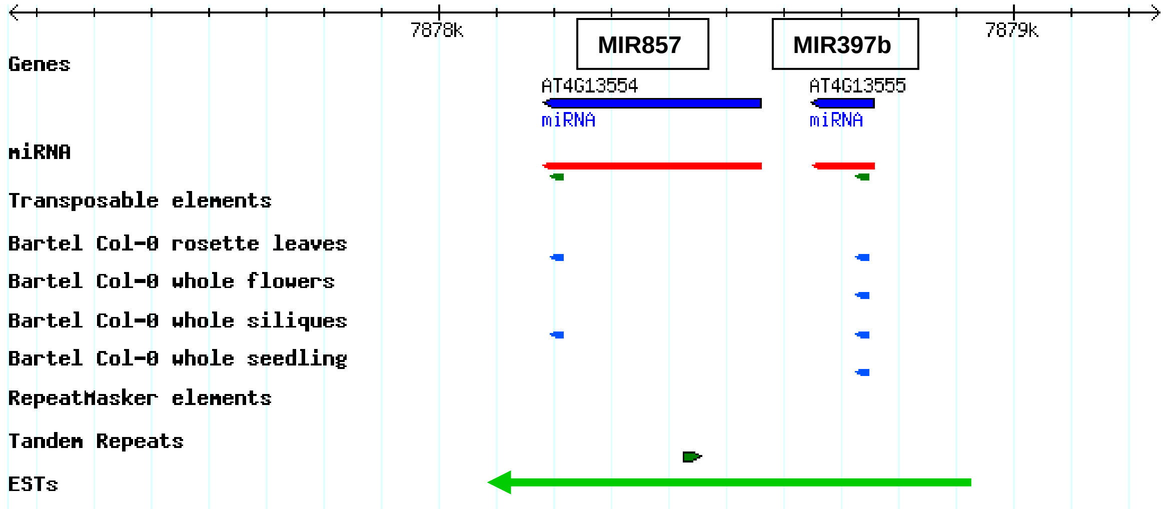

Color key

Seq. Length 19 | Seq. Length 20 | Seq. Length 21 | Seq. Length 22 | Seq. Length 23 | Seq. Length 24 | Seq. Length 25

Figure S4 (continued). Small RNA hits in *Arabidopsis* polycistronic non-homologous miRNA clusters (based on the "Genome View" browser in ASRP database; http://asrp.cgrb.oregonstate.edu/) Green arrows represent polycistronic ESTs, encompassing both predicted miRNA hairpins, which were amplified by RT-PCR in this study.

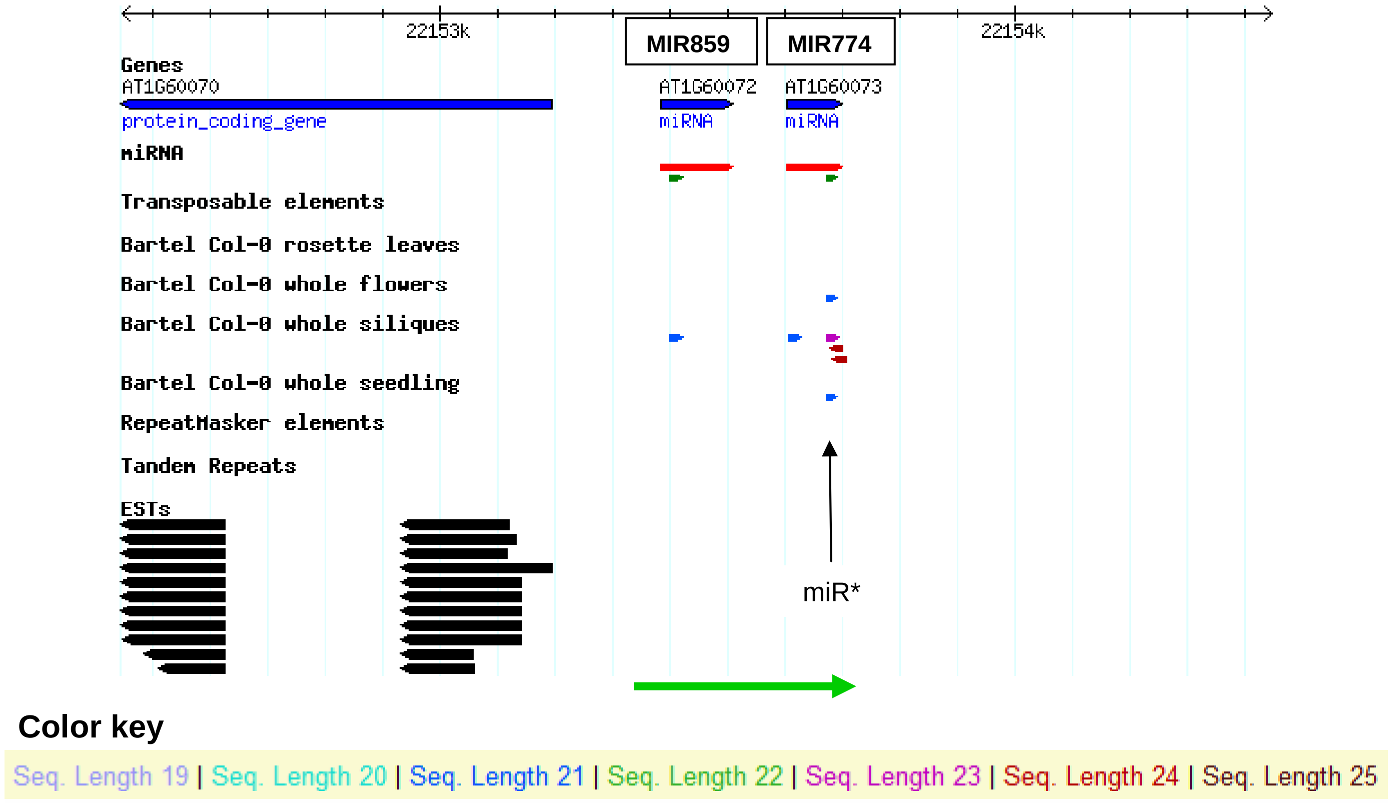

Figure S4 (continued). Small RNA hits in *Arabidopsis* polycistronic non-homologous miRNA clusters (based on the "Genome View" browser in ASRP database; http://asrp.cgrb.oregonstate.edu/). Green arrows represent polycistronic ESTs, encompassing both predicted miRNA hairpins, which were amplified by RT-PCR in this study.
